# Botulinum Toxin A for Elbow Flexor Spasticity: A Non-Randomized Observational Study of Muscle-Specific Injection Strategies

**DOI:** 10.3390/jcm14113864

**Published:** 2025-05-30

**Authors:** Miruna Ioana Săndulescu, Delia Cinteză, Daniela Poenaru, Claudia-Gabriela Potcovaru, Horia Păunescu, Oana Andreia Coman

**Affiliations:** 1 Doctoral School, Carol Davila University of Medicine and Pharmacy, 020021 Bucharest, Romania; 2 Department of Pharmacology and Pharmacotherapy, Carol Davila University of Medicine and Pharmacy, 020021 Bucharest, Romania; 3 Rehabilitation Department, Carol Davila University of Medicine and Pharmacy, 050474 Bucharest, Romania

**Keywords:** neurorehabilitation, botulinum toxin, post-stroke spasticity, elbow flexors

## Abstract

**Introduction:** Elbow flexor spasticity is a common and debilitating consequence of stroke, significantly impacting patients’ quality of life. Botulinum toxin A (BoNT-A) injections have emerged as an effective treatment, but the optimal muscle selection strategy remains unclear. This study investigates the impact of different BoNT-A injection strategies targeting specific elbow flexor muscles in post-stroke patients. **Materials and Methods**: A non-randomized observational study was conducted on 52 participants with upper limb spasticity (pattern IV) following a stroke. Participants were divided into three groups based on the elbow flexor muscles injected with BoNT-A: biceps brachii (*n* = 15), brachialis (*n* = 9), and brachialis plus brachioradialis (*n* = 28). Assessments included spasticity angle, paresis angle, and active supination range of motion (ROM) measured using the Tardieu Scale and goniometry at baseline and at 4-week follow-up. Non-parametric statistical analyses were employed to compare outcomes between groups. Results: While all groups showed a general trend of decreased spasticity and improved motor control, analysis revealed statistically significant differences across the groups at baseline. The brachialis plus brachioradialis group demonstrated the most substantial improvement in paresis angle and active supination ROM. Notably, this group also exhibited greater capacity for the improvement of the paresis angle. The biceps brachii group showed comparable improvements in the paresis angle and the greatest effect on improving passive extension at slow velocity with increasing stroke onset but required higher pronator teres BoNT-A doses overall. **Discussion**: These findings suggest that individualized muscle selection strategies are crucial in BoNT-A treatment for elbow flexor spasticity. The superior outcomes observed in the brachialis plus brachioradialis group may be attributed to the synergistic action of these muscles in elbow flexion and forearm positioning. The higher pronator teres BoNT-A doses required in the biceps brachii group may reflect compensatory mechanisms or differences in muscle fiber recruitment patterns. **Conclusions**: Combining brachialis and brachioradialis muscles in BoNT-A injections appears to offer superior benefits for supination and motor control in post-stroke patients with elbow flexor spasticity, particularly those with significant elbow flexion and pronation.

## 1. Introduction

Spasticity, a velocity-dependent increase in tonic stretch reflexes, is a common and often debilitating consequence of stroke, affecting a significant proportion of chronic post-stroke patients and substantially impacting their quality of life [1,2,3,4,5,6,7]. Upper motor neuron syndrome manifests as increased muscle tone, hyperreflexia, and involuntary muscle contractions [8,9,10,11,12], which in time lead to functional limitations and reduced independence. Within the spectrum of upper limb spasticity patterns, pattern IV [13], characterized by elbow flexion, wrist flexion, and finger flexion, is the most frequent presentation in post-stroke patients [14]. The spasticity of the upper limb is a crucial element in limiting patients’ ability to perform essential daily activities such as reaching, grasping, and manipulating objects, while also contributing to pain, contractures, and the learned non-use of the affected limb [1,2,3,5,6,11,12,15]. The resulting functional impairment can significantly hinder rehabilitation efforts and negatively impact overall quality of life [16].

Botulinum toxin type A (BoNT-A) injections have emerged as a well-established and effective treatment for focal spasticity, offering targeted muscle relaxation and improved function [17,18,19,20,21,22]. By selectively weakening hyperactive muscles, BoNT-A can reduce spasticity-related symptoms and pain [23], improve range of motion, and facilitate participation in rehabilitation therapies. In addition to its neuromuscular blocking effect, BoNT-A–facilitated recovery may also engage broader physiological processes such as vascular remodeling and local tissue adaptation, mechanisms that have been described in other therapeutic contexts involving VEGF-mediated neovascularization [24].

Regarding the spasticity at elbow level, the input of each individual flexor muscle—biceps brachii, brachialis, and brachioradialis—can differ, and it is a widely analyzed and discussed topic, yet there remains no definitive answer in terms of more tailored approaches to BoNT-A injection. While common targets include these primary elbow flexors, achieving optimal outcomes requires the careful assessment of muscle activity and synergistic patterns [13,25,26,27]. Research studies offer varying perspectives on the primary muscle contributing to elbow flexor spasticity. Some studies suggest that the biceps brachii plays the dominant role, citing its biarticular function (crossing both the shoulder and elbow), thus exacerbating spasticity in certain arm positions [28]. Conversely, other researchers emphasize the importance of the brachialis muscle, contending that, because it is the only elbow flexor independent of shoulder movement (monoarticular), it is the most consistent and direct contributor to spasticity at the elbow joint, regardless of the shoulder. This perspective suggests that targeting the brachialis is crucial for achieving consistent reductions in elbow flexion tone. Finally, some evidence points to the brachioradialis as a significant contributor, particularly in cases where spasticity is more pronounced during active movement or in specific forearm positions [27,29]. The brachioradialis’s role as a dynamic stabilizer and its influence on forearm rotation may explain its contribution to spasticity in these scenarios. Understanding these varying perspectives and the underlying arguments is crucial for developing effective and individualized BoNT-A treatment strategies. This study aims to contribute new data to the existing literature, offering insights into effective BoNT-A treatment strategies for elbow flexor spasticity by comparing the effects of different muscle selection strategies for BoNT-A treatment. This study assesses whether varying the choice of injected elbow flexor muscles leads to differential outcomes in terms of elbow spasticity, motor control, and overall functional performance.

## 2. Materials and Methods

### 2.1. Study Design

This clinical observational study was conducted at the tertiary care neurological rehabilitation clinic of the National Institute of Rehabilitation in Bucharest, Romania between January 2019 and December 2023. Participants were selected from the clinic’s inpatient population during this period, based on the fulfillment of the inclusion criteria and the provision of written informed consent. The study protocol was approved by the institution’s Ethics Committee on Human Research [Ethics Committee on Human Research of the National Institute of Rehabilitation in Bucharest, Romania, approval code 1/07.01.2019, approval date 7 January 2019], and all procedures were carried out in accordance with the Declaration of Helsinki. The study employed a prospective, non-randomized observational design. The non-randomized approach was deemed necessary because botulinum toxin A is currently considered the pharmacological treatment of choice for post-stroke focal spasticity, and withholding this treatment from a control group would have been inconsistent with established ethical guidelines [30].

### 2.2. Participants

#### 2.2.1. Patient Selection

**Figure 1:**
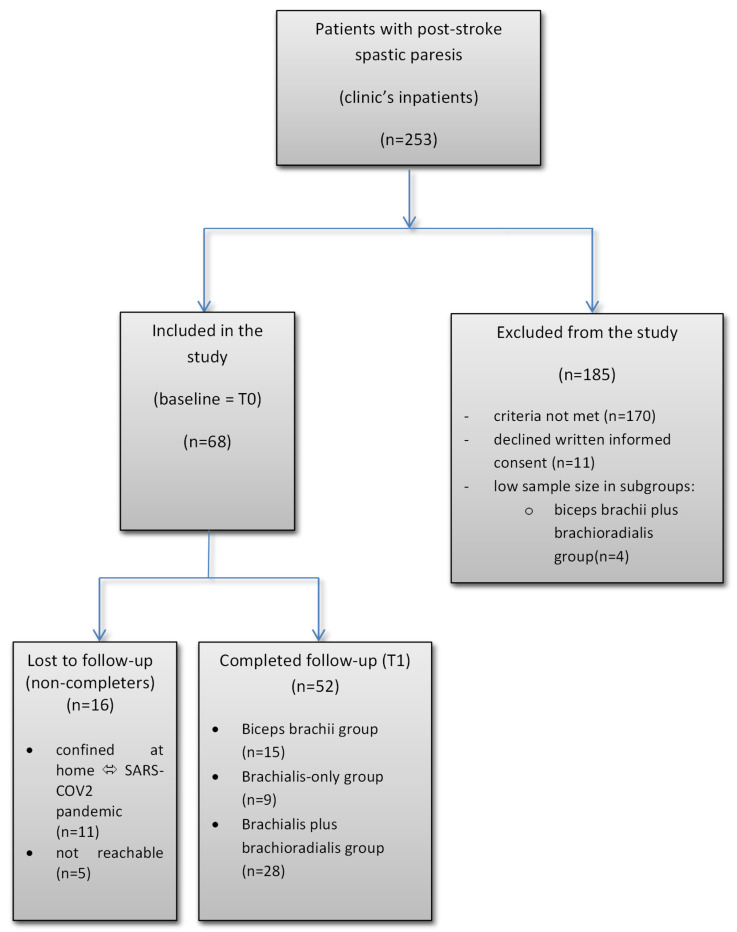
Flow diagram of patient selection for the study.

The study population comprised stroke inpatients from our clinic who fulfilled the inclusion criteria, resulting in a final sample of 52 participants who completed both baseline and follow-up clinical assessments (Figure 1). The patient population included in this study reflects a representative clinical sample of patients who typically require repeated inpatient rehabilitation interventions following stroke.

The flow diagram in Figure 1 presents the number of patients at each stage of the study. A total of fifty-two participants were enrolled in this study and subsequently divided into three treatment groups. While acknowledging the relatively small sample sizes in some groups, we believe that the overall sample size was sufficient to detect meaningful effects, given the effect sizes observed in previous research on BoNT-A treatment for spasticity [31].

#### 2.2.2. Inclusion Criteria

1. Patients who provided written informed consent;
2. Aged ≥18 and ≤80 years;
3. Hemiparesis due to a single stroke occurred ≥2 months before the assessment;
4. Presence of muscle hypertonia at elbow level;
5. Clinical assessment performed just before (T0 = baseline, at the moment of inpatient hospital admission) and after BoNT-A treatment (T1 = follow-up evaluation at 28 days ± 5 days, after hospital discharge) which included the following: (a) motor control at elbow level; (b) spasticity and paresis angle; (c) muscle tone elbow flexors and pronator teres muscle; and (d) active ROM supination angle.

#### 2.2.3. Exclusion Criteria

1. Recurrent strokes or other medical conditions in addition to stroke likely to interfere with the clinical assessment reported in the inclusion criteria;
2. Use of intrathecal baclofen [32];
3. BoNT-A or other neural-blocking agents [33,34,35] injected in the upper limb three months before assessment at T0;
4. Patients with documented or reported medical history of adverse effects following previous BoNT-A injection (e.g., myalgia, muscle weakness, asthenia, flu-like syndrome, local reactions at the injection site, etc.).
5. Severe cognitive impairment;
6. Severe aphasia interfering with patient’s assessment;
7. Degree of spasticity 1 or 4 on MAS (Modified Ashworth Scale);
8. Severe tendon contractures, heterotopic ossification, history of elbow arthrodesis, or other conditions causing secondary limitation of elbow joint mobility;
9. Patients who refused to provide written informed consent.

Of the 253 patients screened, 68 were enrolled. A total of 185 were excluded due to not meeting inclusion criteria (*n* = 170) or declining consent (*n* = 11). Although we evaluated the three main elbow flexor muscles both individually and in combination, a dedicated brachioradialis-only group could not be established, as clinical assessment revealed no cases in which BoNT-A was indicated exclusively for brachioradialis (*n* = 0). In nearly all such cases, other elbow flexors also exhibited spasticity requiring treatment. Additionally, a small subset of patients (*n* = 4) could not be retained due to insufficient representation in the biceps brachii plus brachioradialis group. During the follow-up period, 16 participants were lost to follow-up (non-completers), with 11 confined at home due to the SARS-CoV-2 pandemic [36] and 5 being unreachable. The final analysis included data from 52 participants who completed the study protocol. The fifty-two completers included in the data analyses were divided into three groups according to the elbow flexor muscles injected with BoNT-A: biceps brachii group, brachialis group, and brachialis plus brachioradialis group.

Table 1 presents the demographic and clinical characteristics analyzed across the three groups. The mean age of participants ranged from 53.4 to 59.6 years, with the proportion of females across the three groups ranging from 20.0% to 50.0%. The time since stroke onset varied considerably, with group means ranging from 38.9 to 64 months. Ischemic stroke was the predominant etiology in all groups, although the proportion of hemorrhagic stroke varied (11.1% to 40.0%).

### 2.3. Assessment and Procedure

The primary objective of this study was to assess the impact of BoNT-A (abobotulinum toxin A) treatment on the elbow spasticity angle using the Tardieu Scale [37] by targeting the elbow flexor muscles (biceps brachii, brachialis, brachioradialis) and the pronator teres muscle. Secondary objectives included evaluating the effects of BoNT-A on the elbow paresis angle using the Tardieu Scale and active range of motion (ROM) in supination using goniometry, as well as identifying demographic, clinical, and pharmacological factors influencing treatment response. Additionally, a descriptive analysis of the studied variables was conducted.

Measurements for the Tardieu Scale were taken with the patient lying in a supine position [37,38,39]. The resting elbow angle was assessed with the patient standing, and active supination ROM was measured with the patient sitting, with the elbow flexed at 90 degrees. For participants whose forearm was not in full pronation at the baseline or follow-up assessment, the assessor manually adjusted the forearm to full pronation prior to measurement. To measure the catch and release angle in the pronated forearm position, the axis of the standard manual goniometer was placed on the lateral epicondyle of the humerus [38,39]. The stationary arm was positioned in line with the center of the acromion process, while the moving arm was aligned with the styloid process of the ulna [39]. Baseline clinical examination was performed before BoNT-A injection and before the rehabilitation program on the day of hospital admission.

All patients received abobotulinumtoxin A (Dysport^®^ [40], Ipsen, Paris, France) because the hospital where the study was conducted is supplied with that type of toxin. Injections were administered under ultrasound guidance, with muscle selection and dosing tailored individually based on each patient’s clinical presentation (Table 2). To ensure consistency and reduce variability, all assessments were conducted by the same examiner. The choice of target muscles and corresponding BoNT-A doses followed a structured dosing strategy aligned with both Dysport^®^’s [40] official recommendations and international spasticity management guidelines [41,42]. Mid-range doses were selected and adjusted according to the severity and chronicity of spasticity, in accordance with the hospital’s internal protocol for BoNT-A treatment in post-stroke patients. This approach aimed to balance treatment efficacy with safety, adhering to best clinical practices.

The treatment aimed to reduce hypertonia, enhance motor control and passive joint mobility, and alleviate associated disability and pain. Following BoNT-A administration, all patients participated in a standardized 10-day inpatient rehabilitation program, adjusted according to comorbidities and effort tolerance [43]. This program included twice-daily sessions of physical therapy, robot-assisted therapy, and electrotherapy, which are standard components of multidisciplinary post-stroke rehabilitation in our clinical setting. The inclusion of the rehabilitation protocol reflects clinical practice and is routinely used to optimize the effects of BoNT-A by promoting neuromuscular recovery and functional reintegration [44].

The primary endpoint was defined as the change in the elbow spasticity angle between T0 (baseline measurement at inpatient hospital admission) and T1 (follow-up evaluation conducted 28 days ± 5 days after hospital discharge).

### 2.4. Statistical Analyses

Since the data were not normally distributed, non-parametric tests were employed. Normality was assessed using the Shapiro–Wilk test, which indicated a significant deviation from normality for the majority of variables. Therefore, the assumptions for parametric testing were not met, and the Kruskal–Wallis test was used to compare differences between groups. The Wilcoxon signed-rank test was conducted within each group to compare pre- and post-treatment measurements. Statistical analyses were performed using SPSS (Statistical Package for the Social Sciences), version [Amos v20]. Descriptive statistics, including medians and interquartile ranges, were calculated to summarize the demographic and clinical characteristics of the study population. The significance level for all statistical tests was set at *p* < 0.05. Post hoc Mann–Whitney U tests with Bonferroni correction were applied to adjust for multiple comparisons following significant Kruskal–Wallis tests. The strength and direction of associations between continuous variables were examined using Spearman’s rho correlation coefficient. The specific statistical tests used for each analysis are detailed in the Section 3 alongside the corresponding findings.

## 3. Results

Notably, there was a statistically significant difference in the total BoNT-A dose for elbow flexor muscles (*p* < 0.001) across the groups.

Table 3 depicts the spasticity angle, paresis angle, and Modified Ashworth Scale (MAS) scores for elbow flexors and the pronator teres muscles across all treatment groups. When comparing these changes between groups using the Kruskal–Wallis test, statistically significant differences following Bonferroni correction were observed for active supination ROM at baseline and follow-up, pronator teres MAS at follow-up, and the change in pronator teres MAS.

To assess differences in the total BoNT-A dose injected into the elbow flexor muscles, a Kruskal–Wallis test was performed, revealing a statistically significant difference between the groups. Post hoc Mann–Whitney U tests with Bonferroni correction were subsequently conducted to compare each pair of groups, yielding significant *p*-values (Table 3). The brachialis plus brachioradialis group received the highest mean BoNT-A dose, while the brachialis-only group received the lowest.

In addition to the primary outcomes, this study revealed several noteworthy secondary findings that varied across the three treatment groups, including differences in supination angle and the BoNT-A dose required for the pronator teres muscle and total BoNT-A dose for elbow flexors.

Regarding the BoNT-A dose for the pronator teres across the three groups, the Kruskal–Wallis test did not reveal a statistically significant difference (*p* = 0.105). Subsequent pairwise comparisons between each two groups using the Mann–Whitney U test, with Bonferroni correction for multiple comparisons, also failed to reach statistical significance. However, the Mann–Whitney U test comparing the biceps brachii group and the brachialis plus brachioradialis group on pronator teres toxin dose showed a trend towards significance (*p* = 0.033, adjusted alpha = 0.0167), suggesting that patients in the biceps brachii group tended to receive a higher dose of pronator teres toxin. This tendency is also reflected in the mean ranks (27.5 for the biceps brachii group and 19 for the brachialis plus brachioradialis group), which indicate higher ranks for the biceps brachii group. This suggests that further investigation with a larger sample size may be warranted to determine if this trend is statistically significant.

**Figure 2:**
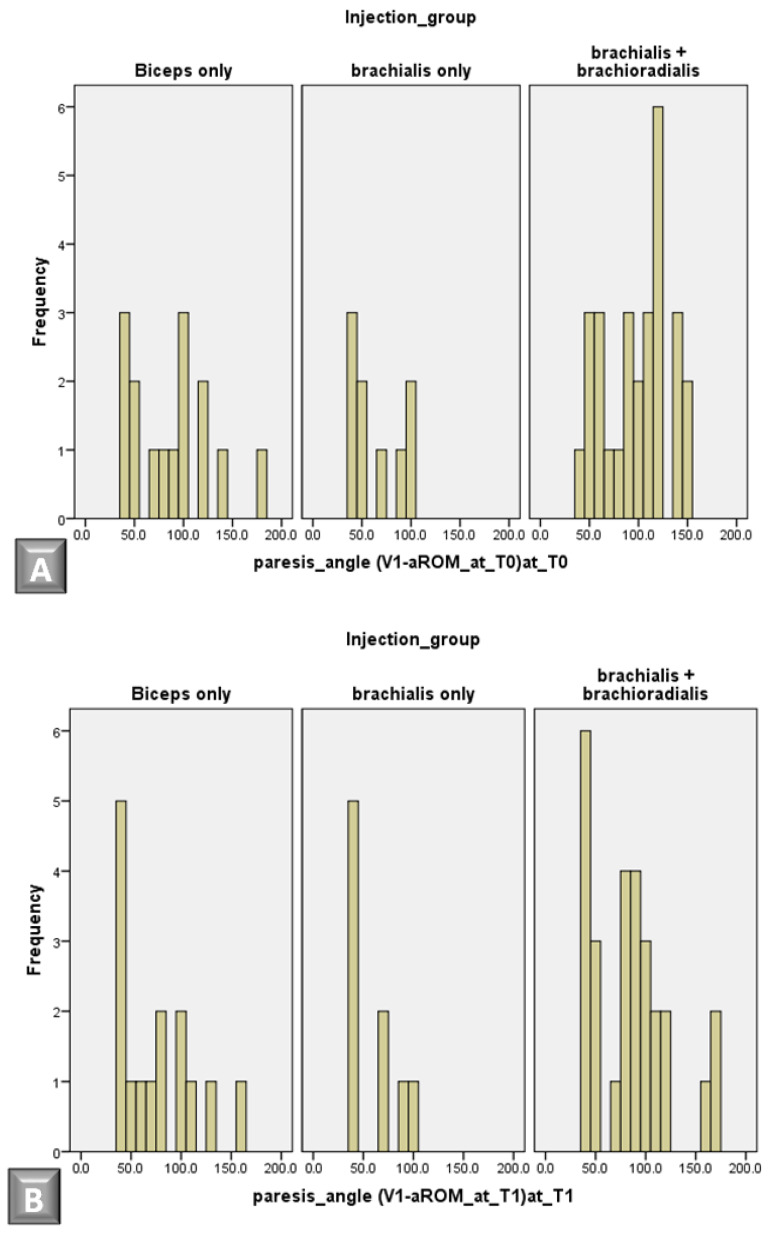

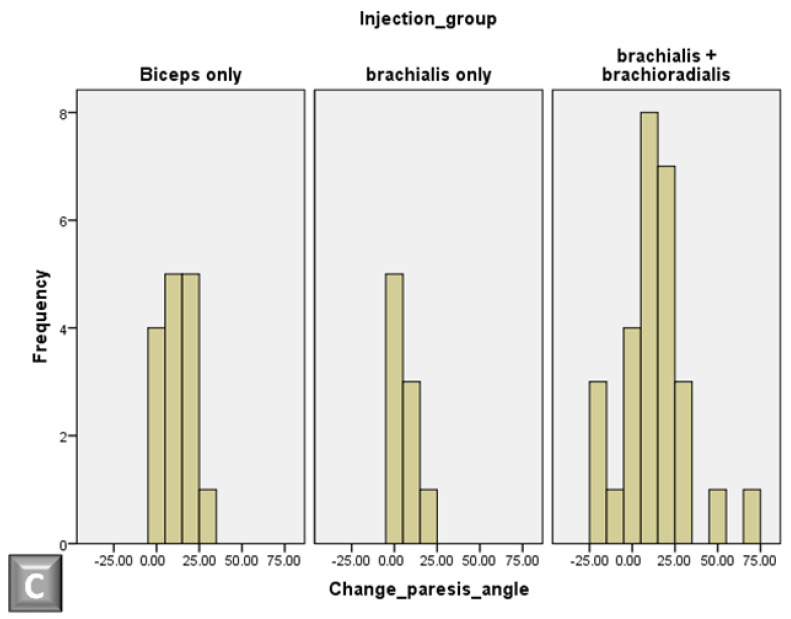
Histograms comparing the distribution of paresis angle across treatment groups at baseline (**A**) and follow-up (**B**) and illustrating the overall change in angle (**C**).

Although no significant inter-group differences were observed in muscle tone (MAS) or spasticity angle (Table 3), Kruskal–Wallis analysis revealed a statistically significant difference in paresis angle across the groups at baseline assessment (*p* = 0.027) (Table 4). Specifically, the brachialis plus brachioradialis group exhibited the highest paresis angle, followed by the biceps brachii group, and the brachialis-only group demonstrated the lowest paresis angle, as indicated by the mean ranks. Furthermore, a statistically significant difference was found between the brachialis-only group and the brachialis plus brachioradialis group at baseline using the Mann–Whitney U test (*p* = 0.007). While the Kruskal–Wallis test did not reveal statistically significant inter-group differences in paresis angle at follow-up, the mean ranks suggest that the greatest improvement in paresis angle occurred in the brachialis plus brachioradialis group (mean rank = 28.08), followed by the biceps brachii group (mean rank = 28.00), with the brachialis-only group exhibiting the least improvement (mean rank = 19.06). Thus, despite having the highest paresis angle at baseline, the brachialis plus brachioradialis group also demonstrated the greatest change (improvement) in paresis angle at follow-up. The biceps brachii group exhibited a similar improvement in paresis angle to the brachialis plus brachioradialis group (mean rank = 28.00 vs. 28.08, respectively), but had a lower paresis angle at baseline (mean rank = 25.00 vs. 30.82, respectively). The brachialis-only group appeared to have the least improvement in paresis angle, as evidenced by its low mean rank at follow-up (19.06), despite having the lowest paresis angle at baseline (mean rank = 19.56). Taken together, these findings suggest that the paresis angle diminished and motor control improved most substantially in the brachialis plus brachioradialis group, followed by the biceps brachii group, with the brachialis-only group exhibiting the least improvement (Figure 2).

The statistical analysis of active supination range of motion (ROM), using the Kruskal–Wallis test followed by Mann–Whitney U tests with Bonferroni correction, demonstrated a significant inter-group difference.

Considering that spasticity angle is calculated as the difference between passive V1 and passive V3, a higher passive V3 is associated with a lower spasticity angle, indicating a more favorable outcome, and also considering the results of the analyses which shows the strongest correlation among the three groups between change in spasticity angle and brachialis plus brachioradialis group, it can be concluded that the latter group is the most effective in improving spasticity at elbow level in regard to the onset of stroke. Of further note is the fact that the biceps brachii appears to be more effective for passive extension at V1, while for active ROM, the biceps brachii and brachialis plus brachioradialis groups show comparable results.

Regarding the change in supination angle, the biceps brachii group exhibited the strongest negative correlation, indicating the least improvement in supination within this group the longer the time since stroke onset. While the brachialis-only group showed limited improvement, the brachialis plus brachioradialis group demonstrated the greatest increase in active supination.

In terms of the change in active extension ROM and, secondarily, the paresis angle, the brachialis-only group demonstrated the strongest correlation, suggesting the greatest effectiveness. However, it is important to note that the brachialis-only group also had the lowest paresis angle at baseline compared to the other groups.

## 4. Discussion

The findings of this study are consistent with the existing scientific literature, particularly in demonstrating improvements in elbow flexor spasticity and motor control following BoNT-A treatment. These improvements were primarily captured through reductions in the spasticity angle and increases in the paresis angle. As shown in Table 3, all groups displayed a general trend of decreased Modified Ashworth Scale (MAS) scores, spasticity angles, and paresis angles for both the elbow flexors and the pronator teres muscle following treatment.

### 4.1. Patient Safety and Selection Considerations

Adverse effects did not occur during the treatment cycle, apart from mild bruising at the injection site and low to moderate procedural pain, both of which resolved spontaneously. To minimize risk and ensure consistency of outcomes, patients with a documented or reported history of adverse reactions to previous BoNT-A injections (e.g., myalgia, flu-like symptoms, or transient muscle weakness) were excluded from the study. This decision was based on both medical records and patient self-reports, aligning with current safety-focused clinical practice.

### 4.2. Forearm Position in Assessment of Elbow Flexor Spasticity

Endorsing previous research [27,28,29,45], the contribution of each of the three main three flexor muscles has been shown to differ. A study on healthy subjects [45] concluded that the muscle which flexes the elbow the most changes with the position of the forearm. Therefore, biceps brachii is attributed the highest importance during forearm supination, brachioradialis during neutral forearm position, while brachialis contributes in all three positions, but primarily in forearm pronation. By employing dynamic electromyography, another study [29] has shown that brachioradialis, followed by biceps brachii, were the most common contributors to elbow spasticity, while brachialis muscle was the least. Integrating both anatomic dissection on human cadavers for the localization of motor nerves supplying the three flexor muscles and, afterwards, performing selective motor nerve blocks on each of them, the study of Genet et al. [28] gathered evidence for brachialis being the most important muscle in post-stroke flexed elbow spasticity. A more recent study [27] performed a clinical assessment on all three forearm positions and concluded that the severity of post-stroke elbow spasticity is highest in pronation and neutral forearm position and least in supination. Consequently, due to the clinic’s unavailability of dynamic electromyography and because the current research involves solely post-stroke patients with spasticity pattern IV [13], we opted for undertaking patient examinations with forearms in pronation.

### 4.3. Selection of Target Muscle

Throughout the years, the procedure for botulinum toxin treatment for focal spasticity in post-stroke patients has not been defined by evenness or consistency, even more so when it comes to selection of the elbow flexor muscles in type IV spasticity patterns [13,46,47,48]. The biceps brachii muscle has been the primary target for the spontaneous flexed elbow in upper neuron disease hypertonia due to a series of characteristics like anatomic visibility and unchallenging approach for invasive maneuvers [17,28,29,45,49], which have served as both an outcome and a catalyst for increased scientific research. Subsequently, studies have emerged regarding the other two main elbow flexor muscles—brachialis and brachioradialis, as well as accessory flexor muscles [50,51] like pronator teres, flexor carpi radialis, and flexor digitorum superficialis; however, there is still no consensus regarding the selection of these muscles for BoNT-A treatment.

From a biomechanical perspective, the biceps brachii [52,53] may not be as advantageous a target for injection in this context, whereas the brachialis [28] and brachioradialis [53,54] are more favorable, and their combination even more so. This could explain why, overall, the biceps brachii group exhibited less favorable outcomes. The change in spasticity angle was similar for the biceps brachii and brachialis groups (Table 4). However, the BoNT-A dose administered to the biceps brachii group was higher than that administered to the brachialis group (Table 2), suggesting that the brachialis is more efficient at lower BoNT-A doses than the biceps brachii. Had the brachioradialis also been injected in the biceps brachii group, the results might have been better, as observed in the brachialis plus brachioradialis group. However, that group received the highest BoNT-A doses. Therefore, we can infer that a higher BoNT-A dose is associated with greater improvements in spasticity, supination, and pronation.

Genet et al. propose that the brachialis is the primary elbow flexor, and that the addition of the brachioradialis may not offer further advantages over targeting the brachialis alone. However, in the present study, this combination appeared to be the most effective treatment strategy. Electromyography studies [29] suggest that the brachioradialis is the most spastic flexor (although co-contraction should be considered) and is highly beneficial for active functional movements, such as reaching. The authors of [53,54] indicate that brachioradialis functions as a flexor and, secondarily, as a pronator, with its flexor activity being most pronounced when the forearm is pronated. Therefore, in cases of significant elbow flexion and pronation, targeting the brachioradialis may be a judicious approach. As shown in Table 4, even though the difference was not statistically significant after Bonferroni correction, the brachialis plus brachioradialis group demonstrated the highest spasticity at baseline (T0), followed by the biceps brachii group, and lastly the brachialis group. This may suggest that (1) this is the reason why BoNT-A dose was highest for this group, and (2) it is possible that in the brachialis group, the inclusion of brachioradialis injections could have yielded improved outcomes.

### 4.4. Primary and Secondary Outcomes: Key Findings and Implications

Although within-group improvements were observed, statistically significant between-group differences after Bonferroni correction were limited to a few key variables. A very strong correlation was observed between both the active range of motion and the paresis angle measured at baseline and at follow-up. This finding contrasts with a previous study’s assertion [55] that arm function does not improve. In our study, based on our testing methods, active ROM increases. However, we cannot definitively conclude whether overall elbow functionality improves, which is a limitation of our study. Furthermore, the aforementioned study [55] suggests that there is no difference in outcomes based on which elbow muscles are injected, stating that the Modified Ashworth Scale (MAS) score improves regardless of muscle selection; however, results from the current analysis yielded several important differences regarding muscle selection.

Another aspect that needs addressing is that within the context of this research, the brachialis plus brachioradialis group received the highest BoNT-A dose. However, as previous research [26] also proposes, considering the potential for high toxin requirements in certain patients, treatment strategies that minimize the total toxin dose (e.g., targeting the brachialis muscle alone) are advisable to prevent exceeding the maximum recommended dose and to address potential limitations in BoNT-A availability. Given the lack of statistically significant differences in paresis angle and spasticity angle between the groups, a treatment approach targeting only the brachialis muscle may be justifiable. Nevertheless, the brachialis plus brachioradialis combination appears to offer greater improvements in supination and necessitates a lower BoNT-A dose for the pronator teres muscle, while the biceps brachii group required the highest pronator teres dose. Nevertheless, it is noteworthy that the total toxin dose for elbow flexors and pronator teres was highest in the brachialis plus brachioradialis group in our study.

Regarding the active range of motion, although the difference was not statistically significant (Table 3), the mean rank order clearly indicates that active ROM increased the most in the brachialis plus brachioradialis group, followed by the biceps brachii group, and then the brachialis group. It appears that the brachioradialis plays a significant role in both active ROM and spasticity angle. Furthermore, it seems that the BoNT-A dose for the brachialis-only group may have been too low. The observed trend aligns with the mean rank order, where the biceps brachii group, which received a higher BoNT-A dose, outperformed the brachialis group. Although the brachialis plus brachioradialis group had a slightly higher mean rank than the biceps brachii group, both groups demonstrated superior outcomes compared to the brachialis group.

### 4.5. Significant Additional Results

**Figure 3:**
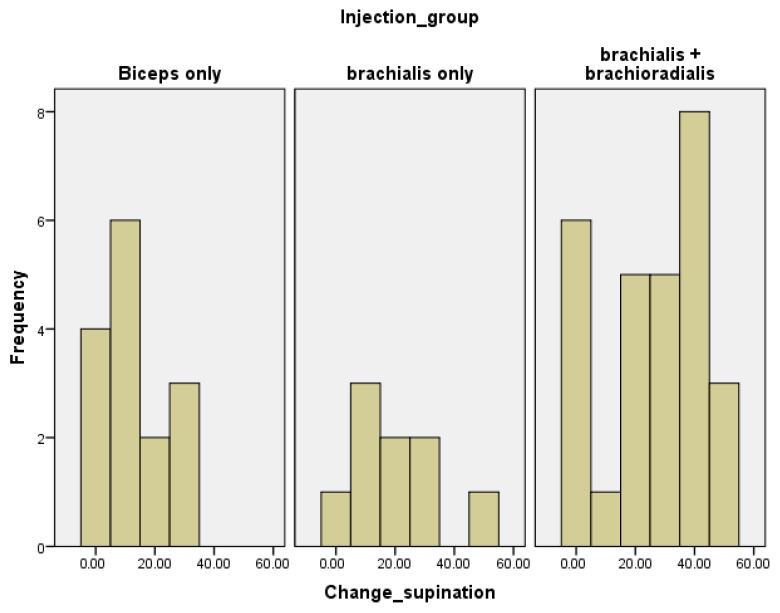
Histogram for change in active supination ROM across groups after treatment.

Among the secondary aspects revealed by the study that are worth highlighting are the significant inter-group differences regarding active supination ROM at both baseline and follow-up, pronator teres MAS at follow-up and the change in pronator teres MAS. Further subgroup analysis demonstrated that the brachialis plus brachioradialis group exhibited the most substantial increase in active supination ROM (Table 4, Figure 3). The statistically significant difference in the supination angle, favoring the brachial and brachial plus brachioradialis groups over the biceps brachii group, can be attributed to several factors already mentioned in the literature. The biceps brachii is a multi-joint muscle that depends on adequate proximal stability of the scapula to function effectively—something often compromised in patients with hemiplegia [28,52]. Due to its structure, the muscle fibers converge into a common distal tendon, resulting in a reduced capacity to withstand stress, which results in a loss of force output. Additionally, the biceps brachii also functions as a forearm supinator, which further limits its role in pure elbow flexion. In contrast, the brachialis is a single-joint, pennate muscle, making it structurally more powerful. Its fibers insert directly into the fascia, enhancing its ability to resist mechanical stress and transmit force efficiently. Therefore, from an anatomical standpoint, the brachialis likely contributes more significantly to elbow flexion than the biceps brachii [28]. Additionally, the brachioradialis muscle also has a more specialized structure for efficient force production in elbow flexion, enhanced by its pennate structure, direct action at the elbow joint, and optimized positioning for power output. While the biceps brachii is a more versatile muscle with actions at the shoulder and forearm, the brachialis and brachioradialis muscles are anatomically designed to be more powerful and focused for elbow flexion. Although not significant after Bonferroni correction (*p* = 0.033), non-parametric tests showed a noteworthy difference regarding the required dose of toxin for the pronator teres muscle (Table 4). Consequently, the highest pronator teres BoNT-A dose was observed in the biceps brachii group and the lowest was identified in the brachialis plus brachioradialis group. This may be attributable to the attending physicians’ established practice patterns in muscle selection, potentially leading to a partial compromise of the biceps brachii’s supination function. This, in turn, could result in increased pronator teres spasticity, which may prompt the administration of a higher BoNT-A dose. However, the absence of data regarding patients’ prior BoNT-A treatments and injection sites is a limitation of this study.

### 4.6. Impact of Stroke Onset on Primary and Secondary Outcome Variables

Spearman’s correlation analysis revealed significant associations between time since stroke onset and several clinical parameters, supporting the hypothesis that spasticity profiles and treatment response may differ by muscle target and stroke duration. Notably, significant correlations were observed for passive and active extension ROM, as well as BoNT-A doses for the elbow flexors and pronator teres muscles.

In terms of the relationship between stroke onset and several primary and secondary outcome variables across the three treatment groups (Table 5), the brachialis plus brachioradialis group demonstrated the strongest correlation with improvements in passive range of motion at V3 (maximum velocity), indicating it was the most effective treatment for elbow flexion. Conversely, the biceps brachii group showed the highest effectiveness for passive ROM at V1 (slow movement), with the brachialis plus brachioradialis group showing moderate efficacy. Biceps brachii muscle could be more effective in passive extension at slow speeds with increasing time since stroke onset due to its ability to adapt to spasticity, its role in muscle coordination and neuroplasticity, and its ability to maintain some function despite the loss of strength in other muscles. Additionally, its eccentric properties and its multi-joint functionality might make it a more resilient muscle for passive movement, especially in the chronic phase of stroke recovery. The analysis also revealed that a higher passive V3, which corresponds to a lower spasticity angle and indicates a more favorable outcome, was observed in the brachialis plus brachioradialis group, demonstrating that this group was the most effective in reducing spasticity at the elbow. Regarding supination, the biceps brachii group showed the strongest negative correlation for changes in supination angle, indicating minimal improvement as time since stroke onset increased, while the brachialis plus brachioradialis group exhibited the greatest improvement in active supination. Finally, for active extension ROM and paresis angle, the brachialis-only group showed the strongest correlation, indicating the greatest effectiveness, although it had the lowest baseline paresis angle compared to the other groups.

### 4.7. Limitation of Study

Several limitations should be considered when interpreting the findings of this study. First, to reduce heterogeneity, outcomes were assessed solely with the forearm in pronation; future research should evaluate results in neutral and supinated positions as well [27,45]. Second, the subjective nature of spasticity assessment using the Modified Ashworth Scale (MAS) introduces a potential source of bias [56,57]. Additional limitations include the unequal number of participants in each treatment group, the lack of quantification of BoNT-A injections administered to wrist muscles, and the absence of data regarding the number of prior BoNT-A injections received by participants. Furthermore, potential practitioner bias related to knowledge, experience, injection technique, and dose selection cannot be excluded. This study also did not quantify structural changes in muscle, such as atrophy or fibrosis. Finally, the single-center design limits the generalizability of the findings, and the relatively small sample size may have limited power to detect smaller, but potentially clinically relevant, effects.

## 5. Conclusions

While our findings support the existing literature regarding the overall effectiveness of BoNT-A in reducing spasticity and improving motor control, as evidenced by the general trend of decreased spasticity angle, paresis angle, and MAS scores across all groups, they also highlight the importance of individualized muscle selection strategies. Several noteworthy secondary findings emerged, including the trend towards higher pronator teres BoNT-A doses in the biceps brachii group, but an overall higher BoNT-A dose for the elbow flexor muscles in the brachialis plus brachioradialis group. Over time, the most significant improvement in motor control and reduction in paresis angle occurred in the brachialis plus brachioradialis group, followed closely by the biceps brachii group. Conversely, the brachialis-only group exhibited the least improvement. Among the treatment groups, post hoc analysis highlighted that patients receiving injections into both brachialis and brachioradialis demonstrated the greatest gains in the active supination range of motion (ROM). Overall, these results suggest that combining brachialis and brachioradialis may enhance recovery more effectively than targeting these muscles individually.

Further subgroup analysis revealed that the chronicity of spasticity—measured by time since stroke onset—was significantly correlated with passive and active elbow extension ROM and the dosage of BoNT-A administered to elbow flexors and the pronator teres. The brachialis plus brachioradialis group in particular showed significant improvement in passive ROM under high-velocity stretch conditions, pointing to greater responsiveness to dynamic muscle lengthening. In contrast, the biceps brachii group exhibited stronger correlations at low velocity, possibly indicating better reductions in baseline tone or static stiffness. The brachialis-only group showed the least improvement overall, which may relate to lower dosing or more limited muscular involvement.

The study demonstrates that although all groups experienced therapeutic benefits, certain muscle-targeting strategies produced more favorable outcomes in specific functional areas. These findings underscore the intricate relationship between elbow flexor spasticity, forearm rotational control, and compensatory muscle activation patterns, reinforcing the importance of comprehensive clinical assessment in guiding individualized treatment approaches.

## Figures and Tables

**Table 1:** Demographic and clinical characteristics analyzed across the three groups.

	Biceps Brachii Group	Brachialis Group	Brachialis Plus Brachioradialis Group	*n* (% of Total)	*p*
Age, year, mean ± SD	59.6 ± 12.2	53.4 ± 11.4	55.6 ± 13.3	-	0.406
Sex, male, *n* (%)	9 (28.1)	5 (15.6)	18 (56.2)	32 (61.5%)	0.889
female, *n* (% within injection group)	6 (30.0)	4 (20.0)	10 (50.0)	20 (38.5%)	
Stroke onset, month	64 (8 to 256)	42 (3 to 223)	38.9 (6 to 123)	-	0.496
Etiology, ischemic, *n* (% within injection group)	9 (60.0)	8 (88.9)	20 (71.4)	37 (71.2%)	0.318
haemorrhagic, *n* (% within injection group)	6 (40.0)	1 (11.1)	8 (28.6)	15 (28.8%)	
total *n* (% within etiology)	15 (28.8)	9 (17.3)	28 (53.8)	52 (100%)	
Affected side, right, *n* (% within injection group)	8 (53.3)	1 (11.1)	10 (35.7)	19 (36.5%)	0.473
Total BoNT-A dose for elbow flexors, mean unit	226 (50 to 400)	122.2 (100 to 350)	319 (100 to 750)	-	<0.001 *
Pronator teres BoNT-A dose, mean unit	143 (50–220)	120 (50–180)	112 (50–200)	-	0.105 *

* *p*-values calculated with pairwise comparisons with adjusted alpha < 0.05.

**Table 2:** Dysport^®^ dosing framework *.

Muscle	MAS 1–1+ (Mild)	MAS 2 (Moderate)	MAS 3–4 (Severe)	Chronicity Adjustment (>6 Months)
Biceps brachii	100–200 U	200–300 U	300–400 U	+10–20%
Brachialis	75–150 U	150–250 U	250–400 U	+10–20%
Brachioradialis	50–100 U	100–200 U	150–250 U	+10–20%

* Extract from the hospital’s internal protocol for BoNT-A (Dysport^®^) administration in post-stroke spasticity.

**Table 3:** Baseline, follow-up, and changed values for elbow spasticity across treatment groups.

	Baseline Assessment						
	Resting Elbow Angle	Passive Elbow Extension ROM at Slow Velocity (V1)	Active Elbow Extension ROM	Spasticity Angle	Paresis Angle	Supination Angle	Pronator Teres MAS
	Mean (SE)	Mean (SE)	Mean (SE)	Mean (SE)	Mean (SE)	Mean rank	Mean rank
Biceps brachii group	136.0 (6.8)	1176.0 (1.9)	128.0 (11.1)	60.0 (6.9)	88.0 (10.6)	23.87	29.77
Brachialis group	151.1 (8.5)	177.7 (2.2)	153.3 (10.0)	45.5 (7.6)	64.4 (8.6)	41.17	17.94
Brachialis plus brachioradialis group	128.2 (4.9)	171.4 (2.2)	112.8 (7.8)	70.3 (4.5)	98.5 (6.2)	23.20	27.50
*p*	0.081	0.169	0.030	0.023	0.027	$0.005^{\;*}$	0.098
	Follow-up assessment						
Biceps brachii group	162.6 (5.6)	180.0 (0.0)	144.0 (9.7)	24.6 (7.0)	76.0 (9.7)	19.70	34.67
Brachialis group	166.6 (4.4)	180.0 (0.0)	161.1 (8.0)	17.7 (7.0)	58.8 (8.0)	40.17	16.22
Brachialis plus brachioradialis group	153.9 (4.2)	178.2 (0.9)	132.5 (7.9)	34.6 (4.8)	85.7 (7.3)	25.75	25.43
*p*	0.119	0.162	0.119	0.179	0.121	${0.005\;}^{*}$	${0.005\;}^{*}$
	Change between assessments						
Biceps brachii group	26.3 (1.8)	4.0 (1.9)	16.0 (2.8)	35.3 (4.0)	12.0 (2.4)	18.77	20.90
Brachialis group	15.5 (6.0)	2.2 (2.2)	7.7 (2.7)	27.7 (4.9)	5.5 (2.4)	25.44	17.56
Brachialis plus brachioradialis group	25.7 (2.1)	6.7 (1.3)	19.6 (3.1)	35.7 (4.8)	12.8 (3.7)	30.98	32.38
*p*	0.105	0.214	0.086	0.484	0.247	0.037	$0.005^{\;*}$

* *p*-values significant after Bonferroni correction.

**Table 4:** Goup comparisons for primary and secondary outcomes.

	Biceps Brachii Group (A) Mean Rank	Brachialis Group (B) Mean Rank	Brachialis Plus Brachioradialis Group (C) Mean Rank	*p* (*a*)	*p* (*b*)		
					(A) vs. (B)	(B) vs. (C)	(A) vs. (C)
Total BoNT-A dose elbow flexors	23.37	9.56	33.63	<0.001	0.003	<0.001	0.015
BoNT-A dose pronator teres	33.27	26.11	23	0.103	0.446	0.433	0.033
Pronator teres MAS change	20.9	17.56	32.38	0.005	0.558	0.012	0.009
Active supination ROM change	18.77	25.44	30.98	0.037	0.263	0.319	0.012
Active elbow extension ROM change	27.1	16.89	29.27	0.086	0.084	0.04	0.597
Paresis angle baseline	25	15.56	30.82	0.027	0.174	0.007	0.243
Change in paresis angle	28	19.06	28.09	0.247	0.123	0.141	0.916

*a*—*p*-values according to Kruskal–Wallis analysis. *b*—*p*-values according to Mann–Whitney U test (adjusted alpha value < 0.05).

**Table 5:** Spearman’s correlation across groups, based on stroke onset.

	Stroke Onset				
	Biceps Brachii Group Correlation Coefficient	Brachialis Group Correlation Coefficient	Brachialis Plus Brachioradialis Group Correlation Coefficient	*p* (Inter-Group Analysis)	Correlation Coefficient (Inter-Group Analysis)
Resting elbow angle	0.336	0.173	0.200	0.008	0.365
Passive elbow extension ROM at V1	0.345	0.137	0.201	0.090	0.237
Passive elbow extension ROM at V3	0.145	0.104	0.385 (*p* = 0.043) *	0.048	0.272
Active elbow extension ROM	0.292	0.347	0.271	0.020	0.321
Active supination ROM	−0.402	−0.128	−0.80	0.144	−0.205
Paresis angle	0.084	0.224	0.168	0.177	0.190
Spasticity angle	0.182	0.097	0.262	0.273	0.155
Total BoNT-A dose elbow flexors	0.327	0.274	0.360	0.11	0.350
Pronator teres BoNT-A dose	0.563 (*p* = 0.029) *	0.288	0.212	0.008	0.361
Pronator teres MAS	0.409	0.156	0.038	0.362	0.129

Spearman’s rho correlations, with correlation significance at the 0.05 level (two-tailed). * *p*-values with statistical significance only, shown in table.

## Data Availability

Data provided upon reasonable request.
